# Neuromodulating the performance monitoring network during conflict and error processing in healthy populations: Insights from transcranial electric stimulation studies

**DOI:** 10.3389/fnint.2022.953928

**Published:** 2022-07-22

**Authors:** Gabriele Fusco, Azzurra Cristiano, Anna Perazzini, Salvatore Maria Aglioti

**Affiliations:** ^1^“Sapienza” University of Rome and CLN2S@SAPIENZA, Istituto Italiano di Tecnologia, Rome, Italy; ^2^IRCCS Santa Lucia Foundation, Rome, Italy

**Keywords:** performance monitoring, conflict and error monitoring, transcranial direct current stimulation (tDCS), transcranial alternating current stimulation (tACS), non-invasive brain stimulation (NIBS)

## Abstract

The performance monitoring system is fundamentally important for adapting one’s own behavior in conflicting and error-prone, highly demanding circumstances. Flexible behavior requires that neuronal populations optimize information processing through efficient multi-scale communication. Non-invasive brain stimulation (NIBS) studies using transcranial magnetic stimulation (TMS) and transcranial electrical stimulation (tES) fields to alter the cortical activity promise to illuminate the neurophysiological mechanisms that underpin neuro-cognitive and behavioral processing and their causal relationship. Here, we focus on the transcranial direct current stimulation (tDCS) and transcranial alternating current stimulation (tACS) that have been increasingly used in cognitive neuroscience for modulating superficial neural networks in a polarity (tDCS) and frequency/phase (tACS) fashion. Specifically, we discuss recent evidence showing how tDCS and tACS modulate the performance monitoring network in neurotypical samples. Emphasis is given to studies using behavioral tasks tapping conflict and error processing such as the Stroop, the Flanker, and the Simon tasks. The crucial role of mid-frontal brain regions (such as the medial frontal cortex, MFC; and the dorsal anterior cingulate cortex, dACC) and of theta synchronization in monitoring conflict and error is highlighted. We also discuss current technological limitations (e.g., spatial resolution) and the specific methodological strategies needed to properly modulate the cortical and subcortical regions.

## Introduction

Our lives are permeated by conflicts and errors. As a matter of fact, what characterizes our behavioral performance is intimately connected with the abilities to process conflicting representations, avoid maladaptive outcomes, and take the most adequate decisions to successfully complete tasks in ever changing environments. But how does the brain perform these sophisticated operations?

For decades scholars from several disciplines debated on the processes and functional executive mechanisms that support flexible behavior under critical circumstances (Theeuwes et al., 2000). However, only with the advancement of neurotechnology in cognitive neuroscience it has been possible to *look at* and *interact with* the activity of neuronal systems involved in performance monitoring and control. So far, neuroscientific evidence confirmed the existence of two complementary cognitive functions that result essential for implementing behavioral adjustments during demanding task performance: the conflict and error monitoring (Botvinick et al., 2001). The neural system selectively activated when conflicts and errors are processed is the Anterior Cingulate Cortex (ACC, Devinsky et al., 1995). The interconnections of the ACC with other cortical and subcortical structures make this area an important computational hub of the neural network involved in the top–down control of behavior (Paus, 2001; Botvinick et al., 2004; Ridderinkhof et al., 2004). Indeed, when the monitoring system detects emerging conflicts in information processing that may lead to errors occurrence, the ACC orchestrates the need to allocate cognitive resources to task-relevant features and optimize flexible adjustments (Luu et al., 2004; Cohen and Donner, 2013). Neuro-electrical signatures of this process are typically recorded over the medial frontal sites of the scalp as Event-Related Potentials (ERPs) functionally related to conflict (i.e., the N200; Folstein and van Petten, 2008) and to error monitoring (i.e., the error-related negativity or ERN; the Positivity error or Pe; Falkenstein et al., 1991; Gehring et al., 1995; Falkenstein et al., 2000; Pezzetta et al., 2018, 2021). Importantly, these frontocentral potentials reflect common spectral features in the theta rhythm (4–8 Hz), a further endogenous oscillatory biomarker generated by the ACC (Ishii et al., 1999; Luu and Tucker, 2001) that can be recorded over the midline of the frontal cortex during performance monitoring (Cavanagh and Frank, 2014).

While human studies have provided clear correlative evidence for a link between neuronal activity and monitoring functions, the investigation of their causal relationship still appears far from being exhaustive. This may depend on some limitations of non-invasive brain stimulation (NIBS) techniques, like the transcranial magnetic stimulation (TMS) and direct (tDCS), alternating (tACS) and random-noise (tRNS) electrical stimulation (tES), which are commonly employed in basic and applied research to illuminate the link between brain activity and behavioral performance in a given task. It is well known that by using coils and electrodes placed over the scalp, it is possible to deliver magnetic and low-intensity electrical stimulation to test inhibitory or facilitatory protocols and measure their effects at neurophysiological and behavioral levels. However, targeting the activity of neuronal populations belonging to deep cortical regions, such as the dACC, with a reliable spatial resolution, can result difficult due to constraints related with NIBS parameters and devices. In fact, while TMS targets neuronal activity with a spatial resolution of approximately 0.5–1 cm, tES affects large portions of the superficial cortex depending on electrode size (e.g., 25 or 35 cm^2^). Interestingly, new stimulation protocols using different coil shapes for repetitive TMS (e.g., H-coils) or electrode arrangements for tES (e.g., high-definition) seem to be promising for targeting deep brain structures. It is worth noting, however, that neurophysiological studies indicate that current NIBS approaches have not an optimal spatial resolution for stimulating deep ventral brain structures (see for example, Hayward et al., 2007 for rTMS-PET co-registration during the Stroop task performance).

**FIGURE 1:**
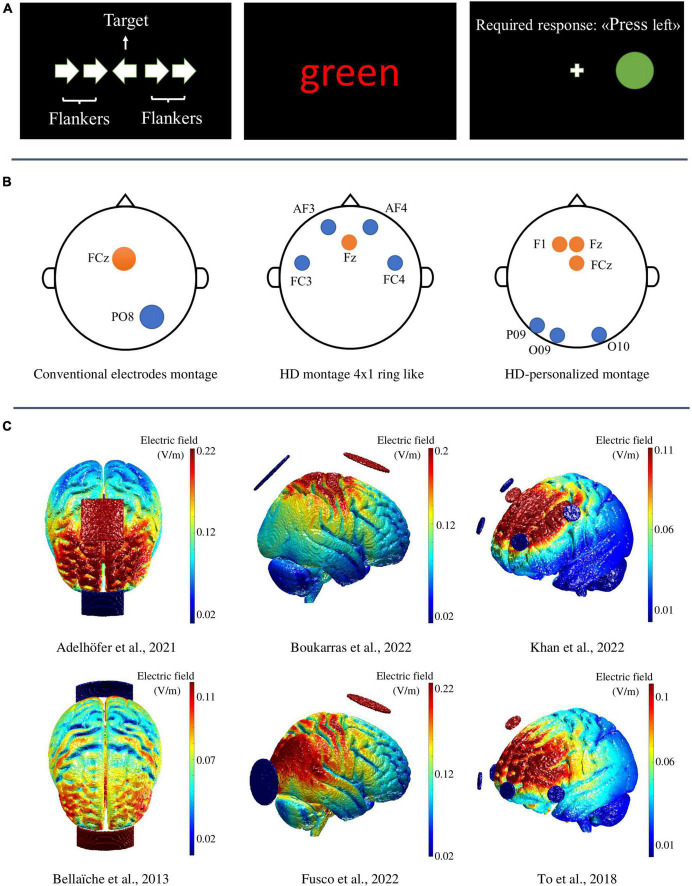
The upper panel **(A)** displays examples of incongruent trials of the Arrow-Flanker, Color–Word Stroop, and Simon task, respectively. In the Flanker task conflict occurs when the flankers are dissimilar from the target (e.g., the flanking arrows point in the opposite direction than the target) and compete for activating different stimulus-motor representations. In the Stroop task, the conflict arises because participants must suppress the involuntary processing of a task-irrelevant attributes (i.e., the meaning of the color word) and attending for less automatically processed task-relevant attributes (i.e., the color in which the word is printed). Finally, in the Simon Task participants are asked to respond to a lateralized target (e.g., by pressing the left button when a green circle appears), irrespective of the effective spatial position of the target. Here, the conflict arises when the spatial position of the target and the response side do not correspond (i.e., the green circle appears on the right). The middle panel **(B)** displays three examples of electrodes’ montage in transcranial alternating current stimulation studies (from left to right, Fusco et al., 2022; Khan et al., 2022; and Mattavelli et al., 2022). The lower panel **(C)** shows three different views of 3D cortical maps representing the electric field intensity distribution (simulated through the open-source software ROAST; https://www.parralab.org/roast/; Huang et al., 2019) of six tES experimental studies differing for both the employed electrodes sizes and montages; from left to right: (i) the axial views of two transcranial direct current stimulation (tDCS) studies both employing a conventional bipolar tDCS montage with two rectangular electrodes of 25 cm^2^ (Adelhöfer et al., 2021) and 35 cm^2^ (Bellaïche et al., 2013); (ii) the saggital views of two transcranial alternating current stimulation (tACS) studies employing a conventional tACS montage with two circular electrodes of 25 cm^2^; (iii) the antero-sagittal views of two HD-tDCS studies both employing a 4 returns × 1 active ring-like montage.

Here, we aimed at reporting recent evidence showing applications of tDCS and tACS used in controlled settings to neuromodulate the performance monitoring network (see Table 1 for the schematic summary of the studies discussed in the review). Specifically, we highlight the strengths and weaknesses of experimental designs that adopt conventional and advanced protocols to target the activity of the medial frontal cortex (MFC) and ACC. However, not all the cortical areas that are strictly connected to the ACC and functionally related to the conflict and error monitoring (e.g., top–down control and response inhibition) are discussed in the present review [see Zmigrod et al., 2016 and Dubreuil-Vall et al., 2019 for some examples of the dorsolateral prefrontal cortex DLPFC modulation during task performance and Cunillera et al., 2016 for the right inferior frontal gyrus (rIFG)]. Since most of the investigations using TMS have been already reviewed in detail elsewhere (see for example, Olk et al., 2015), here we look exclusively at studies delivering tES. Moreover, we focus on classic laboratory tasks (Figure 1A) measuring conflict and error monitoring like the Stroop (Stroop, 1935), the Simon (Simon and Rudell, 1967), or the Eriksen Flanker (Eriksen and Eriksen, 1974). The first section is dedicated to experimental applications of tDCS on cortical regions involved in processing conflict- and error-related representations. The second part describes the behavioral effects of exogenous theta oscillations injected by tACS during task performance. Future developments of neuromodulatory methods will be discussed in the final part.

**TABLE 1:** Schematic summary of the studies discussed in the review and concerning methodological features of the adopted transcranial direct current stimulation (tDCS) and transcranial alternating current stimulation (tACS) protocols.

References	tES technique	Cortical target	Electrodes	Protocol	Parameter	Adverse effects
Bellaïche et al., 2013	Conventional tDCS	Medial prefrontal cortex (mPFC)	2 rectangular (35 cm^2^)	Anodal/cathodal/sham	22 min of DC at 1 mA	No adverse effects are reported
Reinhart and Woodman, 2014	Conventional tDCS	Medial frontal cortex (MFC)	2 rectangular (active, 19.25 cm^2^; return, 52 cm^2^)	Anodal/cathodal/sham	20 min of DC at 1.5 mA	No adverse effects are reported
Adelhöfer et al., 2021	Conventional tDCS	Medial frontal cortex (MFC)	2 rectangular (25 cm^2^)	Anodal/sham	15 min of DC at 2 mA	No adverse effects are reported
Khan et al., 2022	HD-tDCS	Medial prefrontal cortex (mPFC)	4 returns x 1 active ring montage	Anodal/sham	17 min of DC at 2 mA	Some participants reported strong sensation of itchiness or burning during the stimulation.
Mattavelli et al., 2022	Optimized HD-tDCS	Dorsal anterior cingulate cortex (dACC)	3 circular anodes and 3 cathodes (∼1 cm^2^)	Anodal/cathodal/sham	20 min of DC at 1 mA	No adverse effects are reported
To et al., 2018	Optimized HD-tDCS	Dorsal anterior cingulate cortex (dACC)	4 return x 1 active montage	Anodal/cathodal/sham	20 min of DC at 1 mA	No adverse effects are reported
van Driel et al., 2015	Conventional tACS	Medial frontal cortex (MFC)	3 rectangular (1 active: 9 cm^2^; 2 returns: 35 cm^2^)	Theta- and alpha individual frequency-tACS	20 min of AC at 1 mA	No adverse effects are reported
Fusco et al., 2018	Conventional tACS	Medial frontal cortex (MFC)	2 circular (25 cm^2^)	Frequency dependent-tACS (2 Hz/delta, 6 Hz/theta, 11 Hz/alpha, 21 Hz/beta and 60 Hz/gamma and sham)	5 x ∼240 s lasting blocks (20 min in total) of AC at 1.5 mA	No adverse effects are reported
Fusco et al., 2022	Conventional tACS	Medial frontal cortex (MFC), extrastriate body area (EBA)	2 circular (25 cm^2^)	Theta-tACS (6 Hz), gamma-tACS (40 Hz), sham-tACS	∼3 min for each block, for a total of ∼12 min of AC at 2 mA in each session	No adverse effects are reported
Lehr et al., 2019	HD-tACS	Dorsal anterior cingulate cortex (dACC) *via* dorsolateral prefrontal cortex (DLPFC)	4 return x 1 active montage	Theta-tACS (6 Hz), alpha-tACS (9.7 Hz), sham-tACS	20 min of AC at 1 mA	No adverse effects are reported
Klírová et al., 2021	Individulized HD-tACS vs. conventional tACS	Anterior cingulate cortex (ACC) and medial prefrontal cortex	Variable number of electrodes	Theta-tACS (6 Hz), sham-tACS	30 min of AC at 1 mA	No adverse effects are reported
Boukarras et al., 2022	Conventional tACS	Medial frontal cortex (MFC)	2 circular (25 cm^2^)	Theta and beta individual frequency-tACS and sham	∼18 min for each session of AC at 2 mA	No adverse effects are reported
Giller et al., 2020	Conventional tACS	Medial frontal cortex (MFC)	3 rectangular (35 cm^2^)	Theta-tACS (6 Hz), sham-tACS	∼19 min of AC at 2 mA	Tingling, impaired ability to concentrate and fatigue

## The application of polarized direct current to probe spatial and temporal causality of conflict and error monitoring

The non-invasive administration of low-intensity electrical stimulation over the scalp has been increasingly and systematically adopted (Zaghi et al., 2010; Antal et al., 2014) to modulate the activity of neuronal populations in a polarity-dependent fashion (Nitsche et al., 2007; Woods et al., 2016). Indeed, tDCS acts on the subthreshold transmembrane neuronal polarization inducing upregulation (anodal stimulation, atDCS) or downregulation (cathodal stimulation, ctDCS) of the neuronal firing, altering the excitability of target cortical regions located under the electrodes. However, some critical issues concerning the current spreading and spatial resolution may prevent from drawing clear conclusions about the causal nature of areas implicated in cognitive and behavioral processing. On the one hand, factors like the variability of tissues conduction, the orientation of neurons within cortical layers, and the morphology of brain structures (e.g., sulci and gyri), may complicate the control of the current flow in each individual. On the other hand, the bipolar or multi-channel montages, and the application of large electrodes (e.g., 25 or 35 cm^2^) may not allow to modulate the localized cortical portions of the cortex. Nevertheless, thanks to the improvement of computational modeling and methodological procedures (e.g., high-definition tDCS, HD-tDCS), these constraints have been partially overcome. Studies interested in investigating spatial causality by means of tDCS protocols focused on the fronto-medial regions as primary candidates to probe the neural bases of conflict and error monitoring. By implementing tDCS-EEG co-registration, it has been investigated (Bellaïche et al., 2013) whether ERPs reflecting error detection (i.e., ERN) and error appraisal (i.e., Pe) could result altered following anodal, cathodal, or sham stimulation. In a between-subject design, participants received DC for 22 min over the Fpz and Oz scalp positions (bipolar montage with two 35 cm^2^ rectangular electrodes, Figure 1C) while performing a modified version of the Arrow-Flanker. Despite results did not show any behavioral change neither modulations of the ERN nor tDCS conditions, cathodal stimulation compared to sham reduced the amplitude of the Pe component suggesting a functional inhibitory effect of excitability of neurons belonging to the medial prefrontal cortex (mPFC; Bellaïche et al., 2013). Unfortunately, the weight of individual differences, the lack of altered behavioral outcomes consequent to ctDCS, and the application of large electrodes involving wide portions of the cortex may question the actual causal role of mPFC in error monitoring. Encouraging results, however, come from another investigation (Reinhart and Woodman, 2014) showing how 20 min of tDCS at 1.5 mA over the MFC (FCz, active electrode of 19.25 cm^2^, monopolar montage, extracephalic reference electrode of 52 cm^2^) can modulate, in a polarity-dependent fashion, both behavioral and electrophysiological variables. In within-subject cross-over designs participants were asked to perform a two-alternative forced-choice target discrimination task after receiving tDCS and while the EEG activity was recorded. The analysis revealed amplitude increments and decrements of medial-frontal potentials (like the ERN) when atDCS and ctDCS compared to sham were administered. More importantly, these neurophysiological effects were mirrored by bidirectional behavioral changes according to the type of tDCS protocol. In fact, while atDCS reduced, ctDCS increased error occurrence compared to sham. On the same line, Adelhöfer et al. (2021) reported behavioral and neurophysiological effects of atDCS in conflict monitoring during the Simon task performance. In an off-line protocol, participants received 16 min of 2 mA atDCS over the midline frontal regions (i.e., the anodal 25 cm^2^ electrode was placed along the midline and 1.8 cm anteriorly Cz, the reference 25 cm^2^ over the forehead, Figure 1C) and after that, they executed the task during the EEG. Results indicated a worsening of reaction times (RTs) in processing the perceptual-motor interference (acting on the difference between incongruent and congruent trials) that was paralleled by an increase of the N200 amplitude during stimulus–response transition processing. The above-mentioned evidence seems to validate the causal role of the midline frontal structures in conflict and error processing, even if these protocols may suffer from their very limited spatial resolution. HD-tDCS has been introduced as a new sophisticated method to improve the local cortical neuromodulation through a “ring-like” arrangement (Figure 1C), where a single electrode is surrounded by four opposite-polarized electrodes allowing to achieve a focal modulation and keep the electric field distribution within the ring (Datta et al., 2009). A recent pre-post pilot study aimed at testing the effects of HD-tDCS over the frontal midline (Khan et al., 2022) applying 2 mA anodal DC for 15 min and using as configuration, the anode over the Fz scalp position surrounded by four cathodes (AF3, AF4, FC3, FC4, see Figures 1B,C). Eighteen participants were assigned to either the experimental or the sham group and asked to perform the color-word version of the Stroop task before, during, and after the stimulation while the EEG activity was recorded throughout the experiment. Two functional related results were obtained, namely (i) a post stimulation decrement of the Stroop effect in the atDCS group and (ii) a reduction of midfrontal theta oscillations during incongruent trial processing. Even if preliminary, such evidence likely indicates a beneficial effect of atDCS in modulating the mPFC involved in processing representational conflicts and acting on the request of top–down control. Interestingly, by matching HD-tDCS with high standard modeling and targeting procedures, recent studies attempted to reach and modulate the dACC during task performance. Mattavelli et al. (2022), for example, relying on current modeling, optimized the HD-tDCS montage (Figure 1B) and delivered 20 min of 1 mA DC by using three circular, small (∼1 cm^2^) anodes over Fz-F1-FCz and three cathodes over PO9-O9-O10 to target the dACC. Following a within-subject cross-over design, participants were asked to complete behavioral tasks such as the Arrow-Flanker, while receiving one of the three stimulation conditions (i.e., atDCS and ctDCS, and sham) in different days. Results highlighted a reduction of the flanker effect for RTs when participants were previously stimulated with cathodal modulation. Analogously, another study (To et al., 2018), supported by computational simulations showing electric field distribution within the dACC, proposed an electrodes’ montage different from the classic ring configuration (Figure 1C), and applied the active electrode over the Fz site (the anode for atDCS and cathode for the ctDCS) whereas the returns were placed over the frontopolar channels (Fp1, Fp2, F7, and F8). In turn, in a pre-post between-subject design three groups of participants received either anodal, or cathodal, or sham HD-tDCS and were required to perform different tasks like the cognitive and emotional counting Stroop. The EEG in resting-state was further pre-post recorded. Results reported higher power increase in beta frequency in the dACC and faster RTs in the post-stimulation condition for the anodal HD-tDCS group, whereas in the emotional version of the task, the cathodal HD-tDCS group showed faster response times (RTs) and an increase of theta oscillations involving the dorsal and rostral sections of the ACC. Such a dissociation effect may contribute to highlight the differential functional properties of the ACC sub-segments. Overall, the contribution of tDCS in testing the activity of cortical nodes underlying performance monitoring would look promising. By expanding evidence from TMS investigations, the observed neuromodulatory effects confirm the involvement of MFC, mPFC, and dACC as important structures that regulate the information processing during tasks triggering competitive representations and errors (see Table 1 for a summary). Importantly, the functional property that anodal-tDCS induces facilitation and cathodal-tDCS causes inhibition appears inconsistent among these studies (see Fertonani and Miniussi, 2017 for a detailed explanation of the anodal excitation cathodal inhibition (AeCi) hypothesis). Sophisticated protocols and advanced tools are employed in experimental settings to probe spatial causality. However, reaching deep brain areas non-invasively with millimetric and sub-millimetric resolution still represents a challenge which needs further developments.

## Entraining midfrontal theta oscillations through transcranial alternating current to causally modulate conflict and error monitoring

Transcranial Alternating Current Stimulation (tACS) has received increasing attention in the last decade due to the potential of alternating current to interact with the ongoing endogenous and task-related electrocortical activity in a frequency- and phase-dependent manner (Antal and Paulus, 2013). The waveform of the current is typically sinusoidal with a constant alternating shift between positive and negative polarities. The frequency of the stimulation can be applied using all the oscillatory bands that characterize the cortical rhythmic activity. Thus, tACS represents an effective tool for causally testing the correlational evidence of EEG studies and affect neurophysiological, cognitive, and behavioral outcomes. To date, tACS studies investigating conflict and error monitoring mainly delivered theta oscillations along the frontal midline (MF⊖) with the aim of modulating task performance. A first experimental attempt was conducted by van Driel et al. (2015) who used the optimal individual theta frequency to change the behavior during the resolution of visuo-motor spatial conflicts in the Simon task. Participants in two different separate sessions, received either theta- or alpha-tACS over the MFC (active electrode 9 cm^2^ placed between Cz and FCz, two reference electrodes 35 cm^2^ over the cheeks) at 1 mA for ∼20 min following an online protocol. Behavioral results showed a modulation of the congruency sequence effect (a measure of conflict adaptation) during theta- but not alpha-tACS in which RTs were slowed during low conflict trials. However, the lack of the sham condition may make it difficult to determine whether the effects were driven by the oscillatory entrainment or were related to other unspecific factors. In a within subject-design study (Fusco et al., 2018) the independent effects of five tACS frequencies (i.e., 2 Hz delta, 6 Hz theta, 11 Hz alpha, 21 Hz beta, 60 Hz gamma) and the sham, were tested during the administration of AC at 1.5 mA in correspondence of the MFC (the active over FCz and the reference electrode over Pz, both 25 cm^2^). Participants in six different blocks were asked to perform the classical Letter-Flanker in a pseudo-randomized order. Although no modulations emerged on conflict monitoring, 6 Hz-tACS acted on the post-error adjustment by decreasing the RTs requested for responding correctly to congruent trials after error execution. Importantly, such an effect was significantly different respect to the sham. In a follow-up study, Fusco et al. (2022) aimed at exploring whether tACS could modulate theta oscillations underlying the exchange between distal cortical areas involved in perceptual encoding and conflict monitoring that are both called into play for task resolution. Thus, thirty-two participants completed a modified version of the Flanker with conflicting hand stimuli, while receiving in two separate sessions either 6 or 40 Hz-tACS or sham, over the MFC (FCz, circular electrode 25 cm^2^) and over the extrastriate body-area (EBA, PO8 channel, circular electrode 25 cm^2^, see Figures 1B,C). Compared to sham and control gamma-tACS, when participants underwent theta-tACS, their RTs in performing correct trials were faster, a difference that was not found in the Letter-Flanker task. To increase the focality, a couple of investigations employed high-definition protocols to modulate the activity of cortical areas in a frequency-dependent fashion. Lehr et al. (2019), for example, used HD-tACS to alter dACC activity by directly modulating the left dorsolateral prefrontal cortex with a 4 × 1 ring montage in which the active anode was placed over the AF3 position and the four cathodes over F5, Fp2, F2, and AF7. In this study, participants received 1 mA at 6 Hz (experiment1) or 9.7 Hz (experiment 2 for the control alpha-tACS) for 20 min while performing the word color Stroop task. Results showed that only 6 Hz-tACS decreased the interference effect for the RTs. Interestingly, a recent study using 30 min of theta-HD-tACS (6 Hz), computational modeling and individual MRI for personalizing electrode placements, reported behavioral changes in the Stroop task (i.e., higher interference) respect to non-personalized montages (Klírová et al., 2021).

Beyond the classical paradigms, the modulation of mid-frontal regions with theta-tACS can affect the performance in other experimental paradigms investigating action monitoring during interpersonal interactions (Boukarras et al., 2022; Figure 1C) or subliminal and conscious conflict processing (Giller et al., 2020). This may corroborate the functional specificity of MFθ in driving information processing during performance monitoring. Nevertheless, all the described tACS studies lack EEG/MEG measurements and thus come with a strong limitation that prevents from inferring the neurophysiological mechanism underlying the modulatory effects (e.g., the entrainment of endogenous oscillations).

## Final remarks and future directions

Thanks to the techniques exploiting weak electric fields in a non-invasive and controlled manner, we can: (i) interact with the activation of cortical networks, (ii) modulate the communication between nodes, and (iii) shed new lights on the understanding of the neural mechanisms that orchestrate and regulate the behavior. What we observed so far confirm the previous correlative evidence and results from TMS that point to the fundamental role of midfrontal structures and theta oscillations in modulating monitoring functions. Depending on the methodological strategies and protocols, both tDCS and tACS can modulate the neural processes that influence behavioral indices (i.e., RTs) and underpin cognitive interference, conflict adaptation, and post-error adjustment. TDCS studies show effects on the ERPs amplitude and frequency power modulations underpinning conflict and error monitoring. Although promising, tES techniques have strong methodological limitations related to their spatial resolution and direction of current trajectories reaching cortical targets. Targeting the dACC, for example, represents a big challenge as well as removing artifactual activity from endogenous signals when EEG-tACS co-registration is used to investigate neurophysiological and behavioral task-related patterns (Miniussi et al., 2012). Moreover, it is unclear *what* and *where* we are currently neuromodulating during task performance and we are aware that the methodology needs further improvement to reach highly rigorous standards. It is also worth noting that factors like inter-subject variability, neural state-dependency, and blinding procedures may affect behavioral and neurophysiological changes during tES (Nitsche et al., 2007; Palm et al., 2013; Grundey et al., 2018; Rudroff et al., 2020). Of note, attempts to employ H-coils for targeting the frontal network and the dACC during rTMS, seem to have potentially clinical relevance and therapeutic effects on the treatment of psychiatric and neurological conditions such as obsessive-compulsive disorders (OCD, Carmi et al., 2019), depression (Kreuzer et al., 2015), and cocaine addiction (Martinez et al., 2018). Similarly, the delivery of tES on the frontal areas may have the potential to improve symptoms in psychiatric diseases like OCD (D’Urso et al., 2016) and eating disorders (Mattavelli et al., 2019) or neurodevelopmental disorders like ADHD (see Salehinejad et al., 2020 for a recent review). Finally, emerging NIBS techniques, like the transcranial Focal Ultrasound Stimulation (tFUS; Darmani et al., 2022) may provide innovative protocols for non-invasive neuromodulation of cortical and subcortical brain structures. In fact, using ultrasonic mechanical waves at low frequencies, tFUS can reach deep brain structures with a very high spatial resolution causing changes in neuronal excitability without damaging the stimulated tissues. Hopefully, tFUS will be soon used to drive modulatory effects on the dACC, opening a new era for the causal study of the performance monitoring system and for the development of translational treatments.

## Author contributions

GF wrote the first draft of the manuscript. All authors contributed to the manuscript revision, read, and approved the submitted version.

## Conflict of interest

The authors declare that the research was conducted in the absence of any commercial or financial relationships that could be construed as a potential conflict of interest.

## Publisher’s note

All claims expressed in this article are solely those of the authors and do not necessarily represent those of their affiliated organizations, or those of the publisher, the editors and the reviewers. Any product that may be evaluated in this article, or claim that may be made by its manufacturer, is not guaranteed or endorsed by the publisher.

## Funding

This work was supported by the Italian Ministry of University and Research (Progetti di Ricerca di Rilevante Interesse Nazionale Edit. 2017, Prot. 2017N7WCLP) awarded to SMA.
